# PAIN AND LOWER EXTREMITY FUNCTIONAL LIMITATION IN AMERICAN OLDER ADULTS: A SIX-YEAR FOLLOW-UP STUDY

**DOI:** 10.1093/geroni/igae098.2813

**Published:** 2024-12-31

**Authors:** Jaspreet Sodhi, Soham Al Snih

**Affiliations:** Marshall University, Huntington, West Virginia, United States; University of Texas Medical Branch in Galveston, Galveston, Texas, United States

## Abstract

The objective of this study was to examine the relationship between pain and lower extremity functional limitation (LEFL) among American older adults without LEFL at baseline over 6-years of follow-up. Participants (N=1,717) aged ≥ 65 years were from the National Health and Aging Trends Study (2011-2016). Measures included pain, socio-demographics (age, gender, race/ethnicity, and education), depressive symptoms, obesity, and comorbidities. LEFL was defined as limitation in ≥ one of the following five activities (walking 6 blocks, walking 3 blocks, walking up 20 stairs, walking up 10 stairs, or able to get down on knees). General estimation equation model was fitted to estimate the odds ratio (OR) and 95% Confidence Interval (CI) of any LEFL as a function of pain. All variables were treated as time varying except for gender, education, and race and ethnicity. Participants with pain had greater odds (OR=1.52, 95% CI=1.42-1.63) of reporting any LEFL over time than those without pain after controlling for all covariates. Older age and comorbidities were associated with greater odds of any LEFL while married status and high level of education were factors associated with lower odds of reporting any LEFL. In conclusion, pain was a significant predictor of limitations in lower extremity function. Therefore, early assessment of pain and appropriate treatment could prevent future disability and maintain independence in this population.

